# Metallic phase in stoichiometric CeOBiS_2_ revealed by space-resolved ARPES

**DOI:** 10.1038/s41598-018-20351-y

**Published:** 2018-01-31

**Authors:** T. Sugimoto, E. Paris, T. Wakita, K. Terashima, T. Yokoya, A. Barinov, J. Kajitani, R. Higashinaka, T. D. Matsuda, Y. Aoki, T. Mizokawa, N. L. Saini

**Affiliations:** 1 Dipartimento di Fisica, Universitá di Roma “La Sapienza” - Piazzale Aldo Moro 2, 00185 Roma, Italy; 2 Department of Complexity Science and Engineering, University of Tokyo, 5-1-5 Kashiwanoha, Chiba, 277-8561 Japan; 3 Institute for Solid State Physics, University of Tokyo, 5-1-5 Kashiwanoha, Chiba, 277-8561 Japan; 4 Research Institute for Interdisciplinary Science (RIIS), Okayama University, Okayama, 700-8530 Japan; 5 Elettra, Sincrotrone Trieste, Strada Statale 14, Km 163.5, Basovizza, 34149 Trieste, Italy; 6 Department of Physics, Tokyo Metropolitan University, Hachioji, 192-0397 Japan; 7 Department of Applied Physics, Waseda University, Tokyo, 169-8555 Japan

## Abstract

Recently CeOBiS_2_ system without any fluorine doping is found to show superconductivity posing question on its origin. Using space resolved ARPES we have found a metallic phase embedded in the morphological defects and at the sample edges of stoichiometric CeOBiS_2_. While bulk of the sample is semiconducting, the embedded metallic phase is characterized by the usual electron pocket at X point, similar to the Fermi surface of doped BiS_2_-based superconductors. Typical size of the observed metallic domain is larger than the superconducting correlation length of the system suggesting that the observed superconductivity in undoped CeOBiS_2_ might be due to this embedded metallic phase at the defects. The results also suggest a possible way to develop new systems by manipulation of the defects in these chalcogenides with structural instability.

## Introduction

The discovery of superconductivity in BiS_2_-based materials^1^ has stimulated large interest aiming at the search of superconductors with higher transition temperature in this new family of layered chalcogenides^2,3^. Beyond this, the BiS_2_-based systems are also found to have large potential in the field of thermoelectrics^4,5^ due to highly susceptible nature of their structure that can be manipulated by external conditions including chemical and physical pressures^2,3^. The structural susceptibility is related with the defect chemistry of bismuth ion that makes BiS_2_ square lattice highly instable^6^ with the lower energy state being a disordered state characterized by coexistence of different low symmetry structural configurations^7,8^.

There are now several known BiS_2_-based materials with majority of them having a general formula of *RE*OBiS_2_ (*RE* = rare earth element) in which the electronically active BiS_2_ layers are separated by *RE*O spacer layers. This reflects an evident structural similarity of them to the iron-based LaOFeAs superconductors^9^ containing FeAs layers separated by *RE*O spacers. The *RE*OBiS_2_ systems are band insulators and substitution of F for O in *RE*O introduces electron-doping in the active BiS_2_ layers. This gives rise to an electron pocket of Bi 6*p*_*x,y*_ character, appearing at X point of the square Brillouin zone^10,11^.

Among *RE*OBiS_2_ materials, CeOBiS_2_ is peculiar in which Ce appears in the mixed valence state of Ce^3+^ and Ce^4+^. When doped by substitution in the *RE*O layers, Ce(O,F)BiS_2_ shows coexistence of superconductiviy and magnetism at low temperature^12^. The fact that stoichiometric undoped CeOBiS_2_ compound manifests mixed valence^13^ one may expect the extra charge in CeO-layer to dope the BiS_2_-layer, as the case of extrinsic doping by substitution in which extra charge is placed by F in place of O. Such a situation is known to occur in so-called “self-doped” EuFBiS_2_ superconductor^14^ in which Eu appears in mixed valence state with coexistence of Eu^2+^ and Eu^3+^. Very recent observation of superconductivity in stoichiometric CeOBiS_2_ system^15^ may therefore apparently support the analogy with the self-doped systems. However, the interplay between the rare-earth mixed valence and the rare-earth-to-Bi charge-transfer is not that simple. CeOBiS_2_ single crystals with Ce^3+^ and Ce^4+^ mixed valence as well as EuFBiS_2_ single crystals with Eu^2+^ and Eu^3+^ mixed valence are not superconducting although some electrons are introduced to the BiS_2_ layer. Indeed, angle-resolved photoemission spectroscopy (ARPES) data of non-superconducting CeOBiS_2_^16^ and EuFBiS_2_^17^ show absence of the Bi 6*p*_*x,y*_ electron pockets at the Fermi level. This discrepancy indicates that there may be some new physical mechanism active for the charge transfer between the rare-earth and Bi sites. In this context, the recent observation of superconductivity in undoped CeOBiS_2_^15^ is highly interesting and needs further investigations.

Recently, space resolved ARPES is getting known as an important experimental tool to study inhomogeneous materials^18–21^ providing wealth of information on their electronic structure. Here, to address the above question of observed superconductivity without Fermi surface, we have performed space-resolved ARPES on a single crystal of undoped stoichiometric CeOBiS_2_. The ARPES results obtained using submicron beam size reveal that bulk of the CeOBiS_2_ is electronically homogeneous and insulating without any kind of microscale texturing that may be associated with the mixed valence of Ce. Incidentally, we have found metallic phase embedded in the morphological defects and at the sample edges. This metallic phase is characterized by the usual electron Fermi surface pocket at X point, similar to the doped BiS_2_-based superconductors^22–25^. This unexpected result may provide a possible way to understand the observed superconductivity in undoped CeOBiS_2_. In addition of providing a plausible interpretation of superconductivity in undoped CeOBiS_2_, these results may also suggest a possible way to develop new materials by manipulation of defects in instable structures.

**Figure 1:**
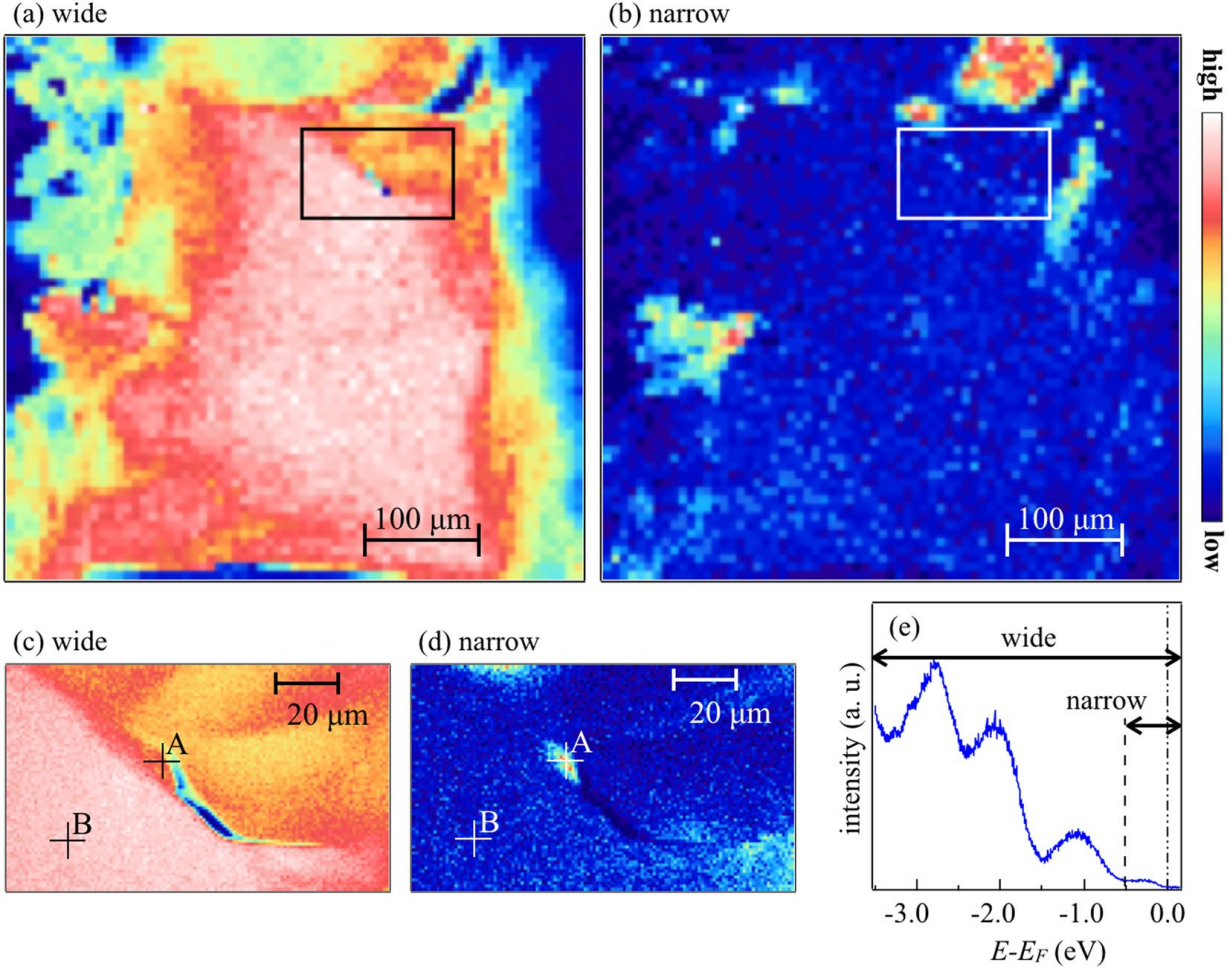
Scanning photoelectron microscopy (SPEM) maps measured on CeOBiS_2_ at 50 K using h*v* = 27 eV. The overview SPEM image is produced by integrating photoemission intensity within the energy interval of $-3.5\,{\rm{eV}}\le E-{E}_{F}\le 0.2$ eV (**a**) and $-0.5\,{\rm{eV}}\le E-{E}_{F}\le 0.2$ eV (**b**). Spatial resolution for the overview SPEM image is 15 × 15 *μ*m^2^. (**c**) and (**d**) are the high resolution SPEM images measured with 1 × 1 *μ*m^2^ resolution (rectangular region of (**a**) or (**b**)). The rectangular region has been chosen considering a defect away from the sample edge. (**e**) Angle- and space-integrated photoemission spectrum. Integrated energy ranges for SPEM images are denoted by ‘wide’ and ‘narrow’ in the photoemission spectrum.

Figure 1 shows scanning photoelectron microscopy (SPEM) maps measured on CeOBiS_2_ sample at 50 K. Fig. 1(a) and (b) represent respectively maps obtained by integrating photoemission intensities within $-3.5\,{\rm{eV}}\le E-{E}_{F}\le 0.2$ eV and $-0.5\,{\rm{eV}}\le E-{E}_{F}\le 0.2$ eV where *E* − *E*_*F*_ represents energy relative to the Fermi level (*E*_*F*_). Apparently, Fig. 1(a) and (b) reveal that majority of the sample is electronically homogeneous within the spatial resolution. However, a clear contrast can be seen in the map obtained by integrating intensity in the energy range of $-0.5\,{\rm{eV}}\le E-{E}_{F}\le 0.2$ eV, indicating that the system contains some inhomogeneity here and there. This contrast (shown by the bright and dark regions in Fig. 1(b)) is most likely due to different phases characterized by different density of states near E_*F*_ since this contrast appears when the integrated range is limited to $-0.5\,{\rm{eV}}\le E-{E}_{F}\le 0.0$ eV. These differences can be better identified in the image shown in Fig. 1(c) and (d), measured with the spatial resolution of 1 × 1 *μ*m^2^ (the region shown by rectangle in Fig. 1(a) or 1(b)). Figure 1(e) shows the angle- and space-integrated photoemission spectrum measured at the center of CeOBiS_2_ sample (majority texture). Three peak structures can be identified in the photoemission spectrum; one is located around −1.1 eV due to Ce 4*f* electrons^16^, and the other two structures are around −2.0 eV and −2.8 eV, mainly due to S 3*p* contributions^10^. The integrated energy ranges are indicated in Fig. 1(e) by ‘wide’ and ‘narrow’.

**Figure 2:**
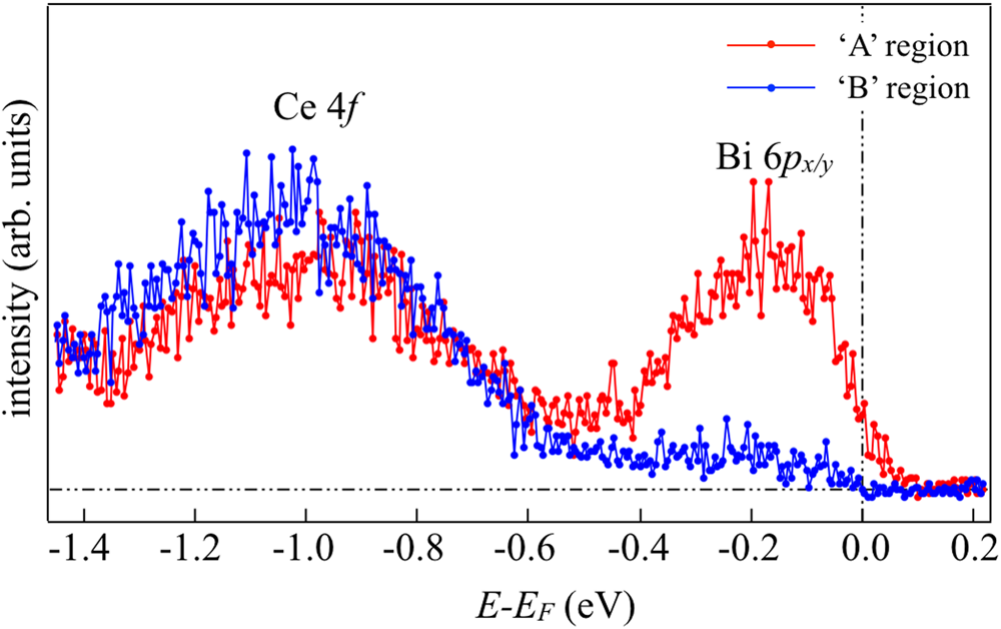
Angle-integrated photoemission spectra measured in the A- and B-points of SPEM maps of CeOBiS_2_ (Fig. 1(c,d)).

The electronic structure of Ce 4*f* in CeOBiS_2_ is similar to what has been measured earlier^16^. It is important to note that the high spectral density phase appears only around the sample edges and around morphological defects. In order to investigate the electronic structure of different phases, we have measured angle-integrated and angle-resolved photoemission (ARPES) spectroscopy in the two phases. These measurements are performed in the region ‘A’ and ‘B’ (in Fig. 1(c,d)) using sub-micron beam size. It is worth mentioning that the region ‘A’ was chosen for ARPES to avoid any possible artefact of sample edge while the integrated spectra were checked to be similar indicating that they should be from the same phase. Figure 2 shows the angle-integrated photoemission spectra measured in A and B points. The electronic structure is substantially different between the two phases. The most important difference is the structure around −0.2 eV, which appears to crosse *E*_*F*_. This difference shows that the two phases seen in Fig. 1(b) and (d) are indeed characterized by very different spectral weight in the vicinity of E_*F*_.

**Figure 3:**
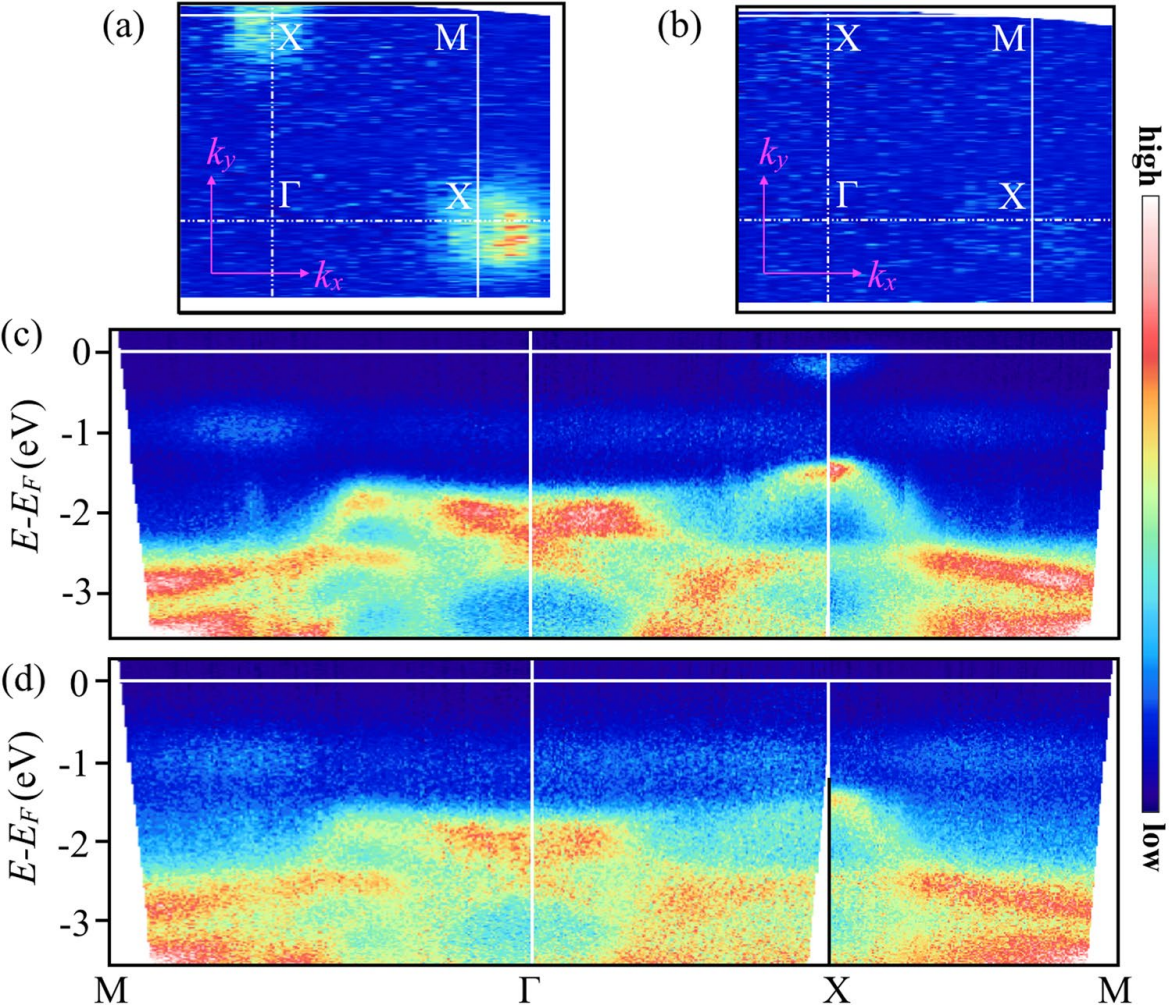
Fermi surfaces for metallic phase at A-point (**a**) and those for semiconducting phase at B-point (**b**) (A- and B-points are indicated in Fig. 1(c,d)). The corresponding band dispersions along M-Γ-X-M are shown in (**c**) and (**d**), respectively.

The next question is the nature of the two phases and if the two are characterized by some dispersive bands and Fermi surfaces. This can be clarified by the ARPES on the two phases measured in the A and B regions. Figure 3(a) and (b) are the Fermi surface maps for the two phases. The Fermi surface maps clearly show that the majority phase (region B) is non-metallic while the minority phase (region A) is metallic. Indeed, the typical Fermi surfaces of the doped BiS_2_-based systems^11,24,25^, characterized by the electron pockets around X point, can be clearly seen in Fig. 3(a) whereas it is absent in Fig. 3(b). The ARPES on the majority phase is consistent with the earlier reports on undoped semiconducting system^16^. The presence and absence of Fermi surfaces in different regions of the sample confirm that the metallic and semiconducting phases are coexisting in stoichiometric CeOBiS_2_. The band dispersions along high symmetry lines of M-Γ-X-M of the Brillouin zone are shown in Fig. 3(c) and (d). As seen in the photoemission spectra (Fig. 2) and the Fermi surfaces (Fig. 3(a) and (b)), the presence/absence of the electron pockets near *E*_*F*_ is the intelligible difference between the two phases. The other features are basically the same except the spectral weight, also seen in the photoemission spectra (Fig. 2). It should be mentioned that no rigid shift has been found in photoemission studies on CeOBiS_2_ system as a function of charge doping induced by F-substitution in place of O^26^. Here, the average shift between different features in Figs 2 and 3 is ∼0.1–0.2 eV, consistent with earlier study on the same system.

Let us discuss briefly possible implications of the present results on the observed metallic phase and possibly the superconductivity in the stoichiometric CeOBiS_2_ system. The space-resolved ARPES results have clearly shown that metallic phase appears embedded in the majority texture of semiconducting phase in CeOBiS_2_. The electronic structure of the metallic phase is characterized by dispersing band structure and Fermi surface pocket around the X point of Bi 6*p*_*x,y*_ nature, typical of doped BiS_2_-based superconducting materials^11,24,25^. Incidentally, the metallic phase is found only around the morphological defects in the crystal while the majority of the sample is highly homogeneous and reveals usual semiconducting characteristics of BiS_2_-based systems without doping or self-doping^10^. Nevertheless, the specific band structure of the metallic phase indicates that this phase is not due to any extrinsic defects but it should be intrinsic to the studied sample. It is also known that, the BiS_2_-based systems are characterized by highly instable BiS_2_ square lattice^7,8^ that makes the properties of these materials highly susceptible to the external conditions including chemical and physical pressures^2,3^. On the other hand, Ce in CeOBiS_2_ appears in mixed valence state with coexisting Ce^3+^ and Ce^4+^ and hence extra electrons are available for charge transfer from the CeO-layer to the BiS_2_-layer.

Here, it should be noted that the Ce mixed valence state is highly homogeneous revealed by space resolved micro X-ray absorption spectroscopy (microXAS)^16^. If the semiconducting region is similar to non-superconducting CeOBiS_2_ and the small metallic region is driven by electron doping due to chemical defects, the Ce valence should be different between the semiconducting and metallic regions and should exhibit inhomogeneous distribution. Therefore, the present observation suggests that the inhomogeneous electronic state of the BiS_2_ layer is not strictly related to the Ce valence. We think that the metallic phase should be stimulated by morphological defects due to change in the local structure around them.

**Figure 4:**
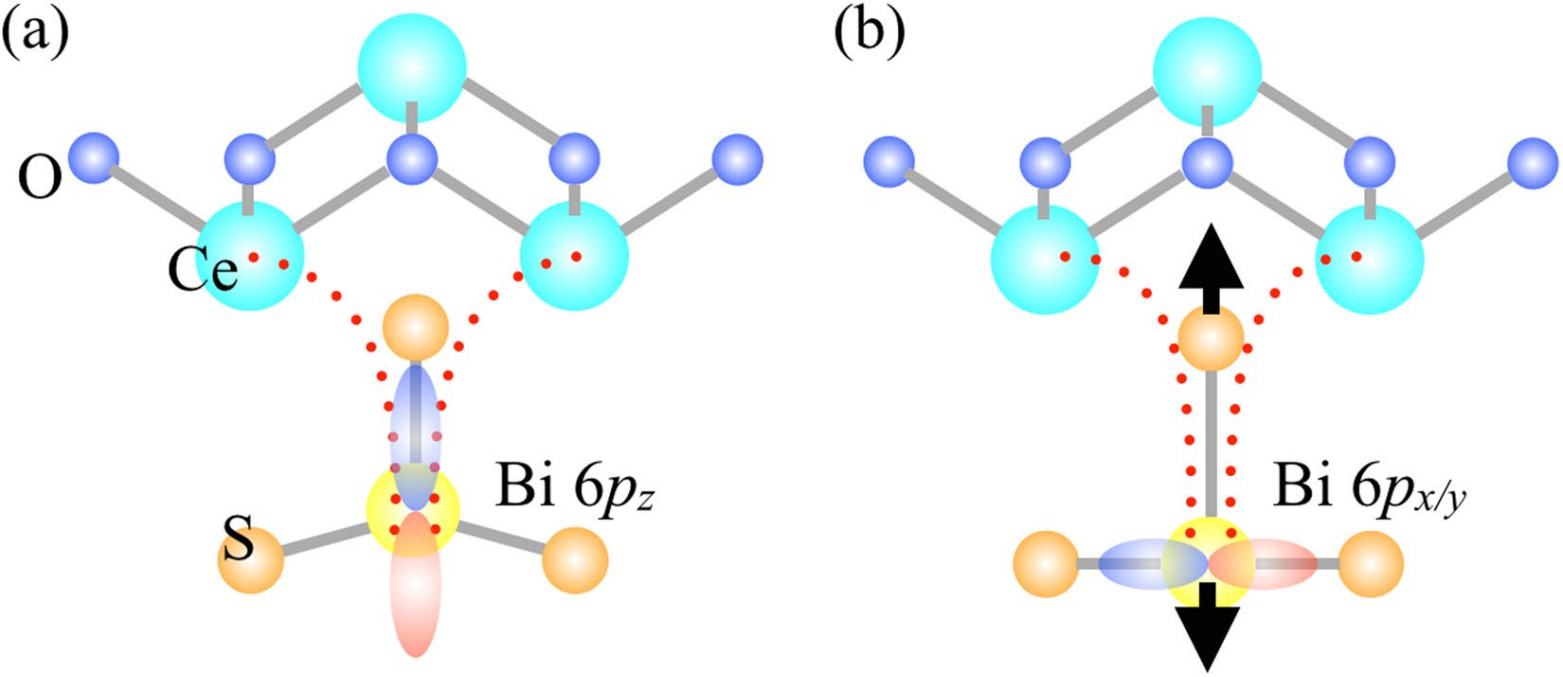
Possible local structure configurations for the semiconducting phase (**a**) and for the metallic phase (**b**) in CeOBiS_2_.

It has been proposed earlier^16^ that the metallic and semiconducting phases have local structure configurations depicted in Fig. 4^13^ and that, in the homogeneous semiconducting region, the self-doped electrons are trapped in Bi 6*p*_*z*_ orbitals due to intrinsic local distortions^13,27–29^ while in the metallic phase they remain mobile in the Bi 6*p*_*x,y*_ due to reduced disorder in the BiS_2_ square lattice. The Bi 6*p*_*z*_ electrons are randomly distributed in the lattice and do not provide a dispersive band. As pointed out earlier, the broad feature within the band gap can be assigned to the Bi 6*p*_*z*_ electrons^16,17^. It is difficult to see exact spectral weight transfer from the Bi 6*p*_*z*_ to Bi 6*p*_*x,y*_ since the former is broadly distributed in the momentum space. Considering all these facts it is plausible to think that the observed metallic phase around the morphological defects (including samples edges) is induced by local strain (in the instable BiS_2_ square lattice) and extra electrons in the CeO-layer (due to mixed valence of Ce). Here it is worth mentioning that although we have put forward a proposal based on structural instability, we are not ruling out completely any peculiar off- stoichiometry or chemical inhomogeneity to drive the metallic phase characterized by energy bands exactly similar to the doped BiS_2_-layer.

Therefore, one possible cause of the recently observed superconductivity in undoped stoichiometric CeOBiS_2_ compound could be the embedded metallic phase in the homogeneous insulating texture. A strong enough intergrain coupling can turn the system into a superconductor at low temperature as in granular superconductors^30,31^. In the studied crystal the volume fraction of the metallic phase is too small for grain coherence to induce bulk superconductivity. In this limit, an insulating behaviour is expected at low temperature at which pairs might have formed locally (locally superconducting) but the pairs remain confined inside the grains (no bulk superconductivity)^30,31^. It should be recalled that the superconductivity coherence length in these materials is less than ∼100 nm^32^ and ARPES with higher space resolution may be helpful to address the exact role of electron inhomogeneity in the superconductivity of these systems.

In summary, we have performed space-resolved photoemission spectroscopy on stoichiometric CeOBiS_2_ system using sub-micron beam size. Using the SPEM imaging we have found a metallic granular phase embedded in the homogeneous semiconducting phase in the undoped system. The metallic phase appears around the morphological defects and is characterized by electron pockets on the Fermi surface, known for the doped BiS_2_-based superconducting materials. We have argued that this metallic phase is formed by the self-doping in the local symmetry broken BiS_2_-square lattice in the proximity of morphological defects. The Fermi surface topology is consistent with the charge-transfer from the mixed valence Ce indicating that the stoichiometric CeOBiS_2_ can be superconducting due to the self-doped carriers in the Bi 6*p*_*x,y*_ orbitals. Therefore, CeOBiS_2_ system, even undoped can show inhomogeneous superconductivity driven by the metallic phase embedded in the insulating texture. The present results may have direct implications on the possible way to develop new materials by manipulation of granular defects in systems with structure instability as the case of Bi-based dichalcogenides.

## Methods

### Sample synthesis and characterization

High-quality single crystals of stoichiometric CeOBiS_2_, prepared by CsCl flux method^33^, were used for the space-resolved ARPES measurements. The sample used for the present work is non-superconducting down to 2 K. The sample is well characterized for its average structure and transport properties and the details are reported in ref.^33^ alongwith the synthesis method.

### Spectromicroscopy measurements

The experiments were carried out at the spectromicroscopy beamline of Elettra synchrotron radiation facility in Trieste, Italy^34^. Linearly polarized light of energy h*v* = 27 eV, focused using a Schwarzschild optics down to 500 × 500 nm^2^ beam spot, was falling at 45° with respect to the flat ab-plane of the single crystal sample for the present measurements. Fermi surface mapping was carried out by changing the position of electron energy analyzer with the photon beam and the sample position fixed. As for the surface treatment of the sample, we cleaved the single crystalline sample at 50 K *in situ* in ultrahigh vacuum (<10^−10^ mbar) in order to obtain a clean (001) surface. The total energy resolution including both monochromator and electron energy analyzer was measured to be ∼100 meV while the angular resolution is ≤0.5 degrees (∼0.021 Å^−1^ in *k*-space). All the measurements were carried out within 12 hours after cleavage and the temperature was kept constant at 50 K.
